# New Progress in Artificial Intelligence Algorithm Research Based on Big Data Processing of IOT Systems on Intelligent Production Lines

**DOI:** 10.1155/2022/3283165

**Published:** 2022-03-10

**Authors:** He Shi, Guohua Cao, Guoqing Ma, Jingsong Duan, Jimeng Bai, Xiangyin Meng

**Affiliations:** ^1^College of Mechanical and Electrical Engineering, Changchun University of Science and Technology, Changchun 130022, Jilin, China; ^2^Chongqing Research Institute of Changchun University of Science and Technology, Chongqing 401135, China; ^3^Wuhu HIT Robot Technology Research Institute CO., LTD, Wuhu 241000, Anhui, China; ^4^College of Mechanical Engineering, Southwest Jiaotong University, Chengdu 611756, Sichuan, China

## Abstract

Intelligent production line is the abbreviation of intelligent production line. Intelligent production line refers to a form of production organization that uses intelligent manufacturing technology to realize the production process of products. The actual manufacturing process includes different levels and links, and each step cooperates to create a high-efficiency production line. The intelligent production line includes 3 levels covering automation equipment, digital workshops, and intelligent factories and runs through 6 major links of intelligent manufacturing (intelligent management, intelligent monitoring, intelligent processing, intelligent assembly, intelligent inspection, and intelligent logistics). The emergence of the Internet of Things system has changed the way of information dissemination. The system combines radio frequency automatic identification and global positioning system technologies to achieve functions such as information exchange and processing, enabling information processing to be intelligent and improving resource utilization. Big data processing includes multiple data processing procedures, but data quality is the most important link in the entire process, and each data processing link will have an impact on the quality of big data. The big data processing process mainly includes data collection, data preprocessing, data storage, data processing and analysis, data display, data visualization, data application, and other links. This article aims to study the new progress of artificial intelligence algorithms for big data processing of IOT systems on intelligent production lines. It is hoped that through the development of intelligent production lines and big data processing technologies, ways to optimize artificial intelligence algorithms can be found. This study proposes a metadata replication method based on a separate replication strategy, which separates the replication process of the data operation log, each is independent, and shortens the data replication time. Combining the existing intelligent production line network platform in the laboratory and carrying out the research of the intelligent production line network state prediction system based on the neural network to design a network prediction system can prejudge the operation status of the intelligent production line network. The experimental results in this article show that when the Namenode mode is used to read data and when the number of clients reaches 8, the data processing basically remains unchanged. When the NCluster system reads data and when the number of clients is 6, the data is processed 1256. When the number of clients is 20, the data is processed 2100, the NCluster system will remain stable when the number of clients reaches 12, and compared with the Namenode system, it has obvious advantages.

## 1. Introduction

The scope of artificial intelligence applications is very wide, including medicine, diagnosis, financial trade, robot control laws, scientific discoveries, and toys. Artificial intelligence, also known as AI, has produced many aspects to solve the most difficult problems in computer science. However, with the ever-expanding social production field, people are increasingly raising production standards, which urgently requires the continuous optimization of artificial intelligence algorithms to reach higher standards. As an important part of the Internet, the Internet of Things can form a new ecosystem of the physical world and the information world, further realize the information sharing of people, machines, and things, make the information exchange between the information world and the physical world closer, and improve social production efficiency and meet people'*s* growing needs. With the increasing popularity of Internet technology, the information data in life have increased exponentially, so the concept of big data came into being. Big data refers to the amount of data involved is so large that cannot be retrieved, managed, processed, and organized in a reasonable time through mainstream software tools to help enterprises make business decisions more positively. How to find the required information from the massive data has become a major challenge for big data processing. With the continuous optimization of big data processing technology, people have been able to select the required information by keywords, which also improves work efficiency and saves time. At present, the development time of artificial intelligence is relatively short, and the algorithm has not been perfected. How to optimize calculation with the help of big data processing and the Internet of Things technology has become a current research hotspot.

Although artificial intelligence is still under development, its future still has many uncertainties, but the rise of artificial intelligence will promote industrial changes and profoundly affect people's lifestyles. There has been a broad consensus that this technology will definitely be a technological revolution with far-reaching impact, so the exploration of artificial intelligence is very necessary. Artificial intelligence technology will have a profound impact on the country's international competitiveness, change the pattern of international industrial division of labor, and reshape the development of the world economy. And focusing on the research of artificial intelligence technology is an important way to improve international competitiveness. In daily life, artificial intelligence technologies such as deep learning, image recognition, and voice recognition have been widely used in smart terminals, smart homes, mobile payments, and other fields to provide ordinary people with wider coverage, better experience, and better convenience in life service.

In view of the characteristics of mass, this article summarizes the related work of the cloud-centric the Internet of Things and summarizes and analyzes the current solutions and research status for the massive data of the Internet of Things. In view of the current research status of the Internet of Things data processing at home and abroad and the analysis of the demand for complex event processing of the Internet of Things, the data processing technology system architecture of the Internet of Things is proposed. Introducing an intelligent recommendation model and using a knowledge base to provide users with a scientific algorithm recommendation plan through the ability of rough set theory to classify knowledge can realize the intelligent recommendation of the algorithm.

## 2. Related Work

Although artificial intelligence technology is a current hot research field, it has a short development time and is not mature enough to integrate with the production field to achieve high-efficiency production. Therefore, how to optimize artificial intelligence algorithms has become a current problem. Yi-Yu systematically proposed the strategies, methods, and engineering theories of intelligent lean manufacturing of traditional Chinese medicine in response to the “six scientific problems” and “five technical challenges” of intelligent manufacturing and lean production of traditional Chinese medicine. The holistic view of traditional Chinese medicine draws on the concept of international advanced pharmaceutical technology and applies the theory and method to six traditional Chinese medicines including Sanqi Panax Notoginseng injection. Based on this theory and method, a number of intelligent production lines have been designed and built, which has greatly accelerated the digital, networked, and intelligent manufacturing of CM. In summary, this theory and application provide a technical demonstration for the technological upgrading and high-quality development of the CM industry [1]. The Internet of Things has a significant impact on the development of manufacturing technology. Therefore, based on the analysis of the challenges and opportunities faced by the manufacturing industry, Liu took the assembly process of mechanical products as the research object and analyzed the characteristics of the manufacturing system based on the Internet of Things. In order to improve the interconnection, perception, efficiency, and intelligence of the assembly system, the author proposed the concept of an intelligent assembly system for mechanical products based on the Internet of Things. Then, introduced the IIASMP framework based on advanced technologies such as information and communication technology, sensor networks, and radio frequency identification. The key technologies such as assembly resource identification, information interaction technology, multisource data perception and fusion, intelligent assembly agent, and data value-added and dynamic adaptive optimization under this framework are described [2]. Wang proposed a new hybrid method that uses an integrated data fluctuation network and multiple artificial intelligence algorithms, called the DFN-AI model. In the proposed DFN-AI model, the author uses complex network time series analysis technology as the preprocessor of the original data to extract fluctuation characteristics and reconstruct the original data, and then uses artificial intelligence tools to model the reconstructed data and predicts the future data. To verify these results, the author checked daily, weekly, and monthly price data from the Cushing Crude Oil Trading Center in Oklahoma. The empirical results show that the proposed DFN-AI model is significantly better than its corresponding single AI model in terms of prediction direction and prediction level. This confirms the effectiveness of the proposed nonlinear model modeling hidden in crude oil prices [3]. Bao uses the Java programming language to design and develop an RPO analysis software based on artificial intelligence (AI) algorithms. The software integrates five different AI algorithms, which can be used for RPO solutions in different situations. Users can quickly select the appropriate RPO calculation algorithm according to the actual situation, and the calculation results can guide the grid company to optimize parameters such as reactive power compensation capacity and transformation ratio. The software mainly includes a project management module, data editing module, RPO calculation module, and control variable editing module. It has a BPA-Matpower data interface, compatible with BPA data format, and can complete the accurate conversion of BPA data to Matpower data. The software can directly edit the BPA data of different grid structures without reprogramming and has the characteristics of high precision and strong versatility [4]. Dou proposed an independent regional connection model in the context of sensor networks to ensure global connectivity and satisfactory data service quality. The author also studied the optimization of deterministic and random deployment of sensor coverage and regional connections in industrial IOT systems. First, a new optimal network that achieves full sensing coverage and ensures regional connectivity is proposed for deterministic deployment. In addition, the optimal model is derived and the advantages of the proposed model are analyzed. Secondly, based on the assumption that a given sensor is deployed as a Poisson point process, a theoretical analysis is proposed to determine the minimum number of sensors for random deployment to achieve a certain coverage and connectivity. Numerical results show that the model is effective for the application of sensor networks in industrial IOT systems [5]. IOT devices are usually small, low-cost, and limited in resources, which makes them vulnerable to physical, side-channel, and cloning attacks. In order to solve this problem, Aman proposed a lightweight mutual authentication protocol based on the physical unclonable function of the Internet of Things system. Two scenarios of protocols are provided, one is when the IOT device and the server want to communicate, and the other is when two IOT devices want to establish a session. The security and performance analysis of the protocols show that they can not only resist different types of attacks but are also very efficient in terms of computing, memory, energy, and communication overhead [6]. Kim proposed an enhanced Access Reservation Protocol (ARP) with a Partial Preamble Transmission (PPT) mechanism for the narrow band Internet of Things (NB-IOT) system. The proposed ARP can improve ARP performance by reducing the occurrence of preamble collisions and is compatible with traditional NB-IOT ARP. At the same time, an analysis model is provided that can capture the performance of the proposed ARP in terms of false alarms, false detections, and collision probability. In addition, the author studied the trade-off between false detection and collision probability and optimized the proposed ARP based on the system load. The results show that the proposed ARP is better than the traditional NB-IOT ARP, especially under heavier system load [7]. Device capture attacks usually threaten the realization of signcryption on unattended devices by extracting encryption keys from the captured devices. Inspired by this problem, Yang proposed a novel and specialized obfuscable aggregatable signcryption scheme (OASC) and signcryption algorithm obfuscator. The design of the scheme considers that the computational and communication costs should be small enough (lightweight), in order to adapt to the applications in resource-constrained embedded devices. The proposed obfuscator can protect the signcryption program from key extraction attacks by converting the program into an incomprehensible obfuscation program [8]. Although these theories have explored artificial intelligence algorithms and big data processing technology to a certain extent, the two fields are less integrated and not practical.

## 3. New Development Methods of Artificial Intelligence Algorithms for Big Data Processing in IOT Systems on Intelligent Production Lines

### 3.1. Big Data Processing

**Figure 1:**
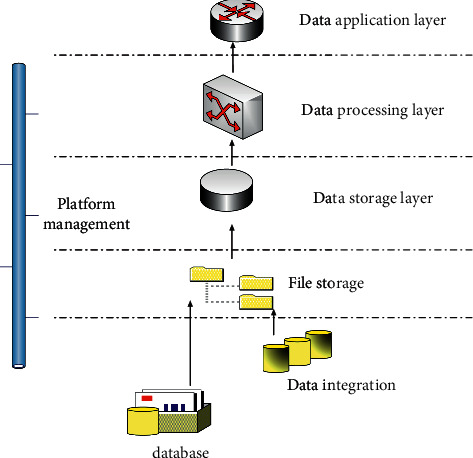
Big data system structure.

Big data brings many problems to data processing. Taking traffic security data as an example, after years of informatization construction, public security, and government departments have collected massive amounts of traffic security-related data resources, the scale and complexity of which have exceeded the management and processing capabilities of traditional databases and software technologies. Since the Internet of Things technology has been promoted, big data is everywhere in daily life, and the scope of application continues to expand [9, 10]. With the emergence of the scale of big data, it is urgently required to classify and process big data. In this context, a big data system has emerged [11], and the structure of the big data system is shown in Figure 1:

The big data processing system mainly includes data integration layer, data storage layer, data processing layer, data analysis and application layer, and platform management, and other components. The data integration layer is responsible for adapting between external data sources and the internal data storage layer of the big data processing system to achieve efficient data import and export.

**Figure 2:**
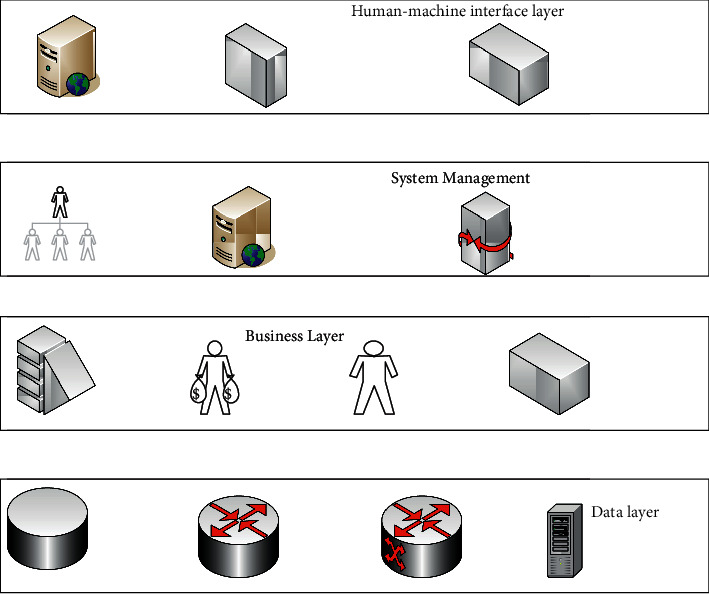
Data preprocessing structure.

With the development of sensor technology, more and more data are collected, prompting the Internet of Things to realize the concept of universal connection to connect every item at any time and place [12, 13]. Using sensors of different specifications to collect data has different data formats, so there will be heterogeneous data, which requires the data to be preprocessed before the data is analyzed [14, 15]. From the original database to the mining database, the operation on the data is called data preprocessing. Preprocessing of data is a complicated process, and different processing programs need to be written for different situations [16]. The data preprocessing structure diagram is shown in Figure 2.

### 3.2. Artificial Intelligence Algorithm

This section mainly discusses related theories about artificial intelligence algorithms, deepens the understanding of artificial intelligence algorithms through the discussion of related theories, and prepares for in-depth discussion [17]:

(1) $$\begin{array}{c} y\left(a|c\right)=\frac{y\left(c|a\right)y\left(a\right)}{y\left(c\right)}=\frac{y\left(c|a\right)y\left(a\right)}{\int y\left(c|a\right)y\left(a\right)da}, \end{array}$$

where *y*(*c|a*) represents the likelihood function, and *y*(*a*) represents the probability density.

(2) $$\begin{array}{c} y\left(c_{1}|a_{2}\right)=\int y\left(c_{1}|c_{2}\right)dc_{2}. \end{array}$$

Formula (2) represents a functional expression for predicting the current probability density function.

(3) $$\begin{array}{c} y\left(c_{1}|a_{2}\right)=\frac{y\left(c_{1}|a_{1}\right)y\left(c_{1}|a_{2}\right)}{\int y\left(c_{1}|a_{1}\right)y\left(c_{1}|a_{2}\right)da_{1}}. \end{array}$$

Formula (3) represents multiple integral operation.

(4) $$\begin{array}{c} W=\int_{S}H\left(a\right)da, \end{array}$$

where *S* is a random area in the space, and W is a multiple integral.

(5) $$\begin{array}{c} W=\int_{S}h\left(a\right)y\left(a\right)da. \end{array}$$

In formula (5), *y*(*a*) ≥ 0 and $\int_{S}y\left(a\right)da=1$.

(6) $$\begin{array}{c} h_{k}=\frac{1}{k}\sum_{1}^{k}h\left(a_{1}\right). \end{array}$$

Formula (6) represents the arithmetic average value.

(7) $$\begin{array}{c} Q\left(1\right)=Q\left(h\left(a\right)\right)=\frac{1}{k}\sum_{1}^{k}h\left(a_{1}\right), \end{array}$$

where *h*(*a*) is an unbiased estimate of *Q*.

(8) $$\begin{array}{c} Q\left(j\left(a\right)\right)=\int j\left(a\right)y\left(a\right)da, \end{array}$$

(9) $$\begin{array}{c} Q\left(j\left(a\right)\right)=\int j\left(a\right)\alpha \left(a\right)\beta \left(a\right)da. \end{array}$$

Among them, *β*(*a*) represents the density function, and *Q*(*j*(*a*)) represents the expected estimate.

(10) $$\begin{array}{c} Q\left(j\left(a\right)\right)=\frac{1}{l}\sum_{1}^{l}j\left(a\right)\beta \left(a\right), \end{array}$$

where *β*(*a*)=*y*(*a*)/*w*(*a*).

(11) $$\begin{array}{c} w\left(a\right)=w_{1}\left(a_{1}\right)w_{2}\left(a_{2}|a_{1}\right)\cdots w_{c}\left(a_{c}|a_{1}\cdots a_{c}\right), \end{array}$$

where a represents a scalar or a vector.

(12) $$\begin{array}{c} y\left(a\right)=y_{1}\left(a_{1}\right)y_{2}\left(a_{2}|a_{1}\right)\cdots y_{c}\left(a_{c}|a_{1}\cdots a_{c}\right). \end{array}$$

In the actual measurement process, each step is independent of each other, so formula (12) represents the density expression of the objective function.

(13) $$\begin{array}{c} \beta \left(a\right)=\frac{w\left(a\right)=w_{1}\left(a_{1}\right)w_{2}\left(a_{2}|a_{1}\right)\cdots w_{c}\left(a_{c}|a_{1}\cdots a_{c}\right)}{y\left(a\right)=y_{1}\left(a_{1}\right)y_{2}\left(a_{2}|a_{1}\right)\cdots y_{c}\left(a_{c}|a_{1}\cdots a_{c}\right)}. \end{array}$$

Formula (13) represents the importance weight.

(14) βjaj=βj−1aj−1yaj−1jwaj−1j,

(15) $$\begin{array}{c} y\left(a_{j}|g_{j}\right)=\sum_{1}^{h}\beta_{j}^{l}\iota \left(a_{j}-a_{j}^{l}\right), \end{array}$$

where *y*(*a*_*j*_*|g*_*j*_) stands for posterior probability.

(16) $$\begin{array}{c} \beta_{j}^{l}=\beta_{j-1}^{l}\frac{y\left(g_{j}|a_{j}^{l}\right)y\left(a^{l}{}_{j}|a_{j-1}^{l}\right)}{w\left(a^{l}{}_{j}|a_{j-1}^{l},g_{j}\right)}. \end{array}$$

Formula (16) represents the approximate posterior probability density of sampled particles.

(17) $$\begin{array}{c} y\left(a|g\right)=\sum_{1}^{h}\beta_{k}^{h}\iota \left(a_{k}-a_{k}^{h}\right), \end{array}$$

where *y*(*a|g*) stands for prior probability.

(18) $$\begin{array}{c} \beta_{j}^{l}=\frac{y\left(a^{h}{}_{j}|g_{k}\right)}{w\left(a^{h}{}_{j}|g_{k}\right)}. \end{array}$$

Formula (18) represents the expression of particle weight function.

(19) $$\begin{array}{c} y\left(a|g\right)=\frac{y\left(g_{j}|a_{1-j},g_{j-1}\right)y\left(a_{1-j}|g_{j-1}\right)}{y\left(g_{j}|g_{j-1}\right)}. \end{array}$$

Formula (19) represents the expression of the posterior probability density function.

(20) $$\begin{array}{c} y\left(a_{j}|a_{j-1},g_{j}\right)=y\left(a_{j}|a_{j-1}\right). \end{array}$$

Formula (20) represents the importance density function in the particle filter algorithm.

### 3.3. IOT System

**Figure 3:**
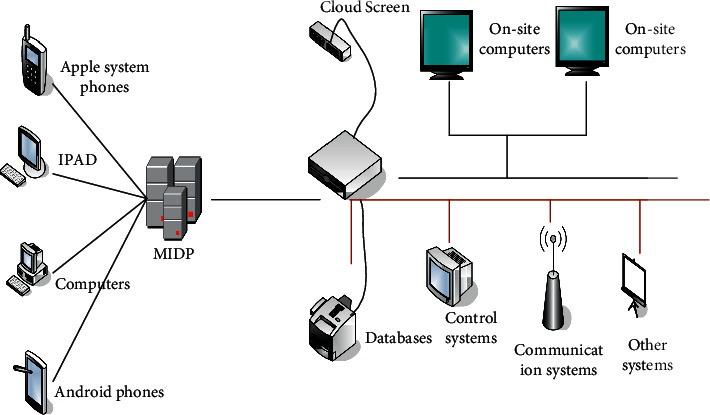
Internet of Things platform architecture diagram.

The Internet of Things is a comprehensive information processing technology based on the network and serving the purpose. It not only makes the way of communication between people leap forward but also makes it possible to communicate between people and things and between things and things. In a word, the Internet of Things technology has turned the whole world into a whole [18, 19]. Networking, IOT, interconnection, automation, perception, and intelligence are the basic characteristics of the Internet of Things. Although the function of the Internet of Things is very powerful, the foundation of the Internet of Things is still the Internet, but the Internet of Things is expanded on the basis of the Internet [20–23]. From the current research progress, the Internet of Things can be divided into perception layer, application layer, and network layer, as shown in Figure 3:

The job of the recognition layer is to recognize the target and record the corresponding information [23]. In this process, network sensor technology or recognizer equipment is involved, and only when multiple technologies cooperate with each other can accurate recognition of the target be achieved [24, 25].

The transportation layer is actually the control backbone of the Internet of Things, which is responsible for the transmission and processing of data information. It includes various corresponding communication networks and networks integrated with the Internet, which are relatively mature parts, such as the Internet, radio and television networks, and so on [26, 27].

The application layer is composed of data collection, monitoring, and energy management. The data collection and monitoring system is to monitor and control the equipment with the computer as the core in order to understand the various data in the system. The management system involves statistics and management functions. All in all, the application layer is the technology that combines the Internet of Things technology with other fields to obtain new intelligent solutions.

**Figure 4:**
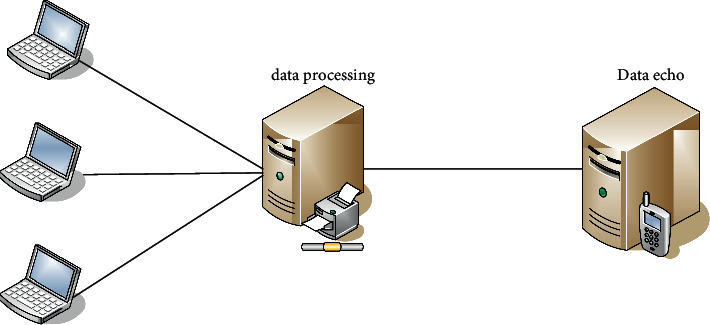
The traditional communication structure of the Internet of Things.

The Internet of Things is a product of the continuous development of information technology. Information technology was first used in the telegraph and telephone. With the continuous innovation of science and technology, the Internet of Things technology has also been continuously developed. The traditional communication structure of the Internet of Things is shown in Figure 4:

The Internet of Things technology is now regarded by workers as the core of the information industry. In the field of the Internet of Things, various elements can communicate with each other and only need to realize information sharing through the Internet of Things technology. The data in the Internet of Things have the characteristics of heterogeneity, mass, and real time. And it has the following characteristics:

1. Transmissibility. The Internet of Things technology can realize the transmission of information by relying on relevant networks and complying with the corresponding network protocols.
2. Comprehensive perception. The Internet of Things is a collection of perception abilities for things and people, it uses technologies such as QR codes to extract information, and it has organizational capabilities, which breaks the dependency of traditional network technologies.
3. Automatic control. Through the use of fuzzy recognition and other corresponding control technologies, related objects can be intelligently managed and used.
4. Intelligent processing. The intelligent processing of the Internet of Things refers to the technology that uses the corresponding data processing technology to process the native data and then transmit it to the user.

**Figure 5:**
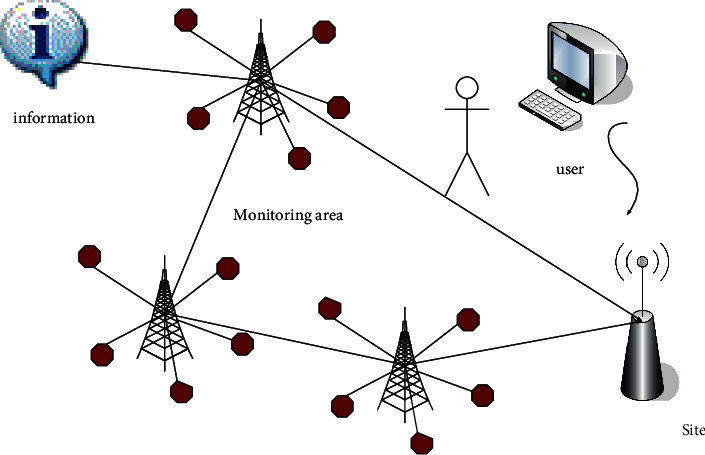
Structure diagram of wireless sensor network.

With the continuous development of science and technology, wireless communication technology has become increasingly mature, and monitoring technology has also been widely used. The early monitoring system used instrument monitoring, followed by a computer monitoring system, which consisted of controlled objects, detection parts, and execution devices. In actual production, some equipment is becoming more and more dispersed, and the independent monitoring of each equipment can no longer keep up with the requirements of monitoring, so a distributed control system appears. The transmission provides centralized monitoring that may follow the needs of social production. Traditional monitoring technology has been unable to adapt, so a distributed control system appears and wireless sensors are produced under this situation. In actual use, multiple wireless sensors are combined to form a wireless sensor network, multiple sensors are placed in the monitoring area to monitor the targets that need to be monitored in real time, and finally, the collected information is systematically analyzed. The core principle of wireless sensor network is that in a specific environment, certain nodes require higher energy, stronger communication distance, and stronger processing capabilities, even including GPS positioning and other functions, and can temporarily act as cluster heads or base stations to provide the function of external network connection. Its specific structure is shown in Figure 5:

By randomly spreading or manually deploying a large number of sensor nodes in the monitoring area, real-time monitoring and collection of the sensing objects that observers are interested in, and then using the node embedded processing system to analyze and process the collected information. The data collected by the nodes in the cluster are aggregated to the cluster head node and then routed to the base station in multihop communication after the cluster head node data is merged. The base station finally transmits the data to the user terminal through wireless or wired means, realizing the user to the remote real-time monitoring of targets.

Currently in the era of information explosion, how to quickly obtain effective information is the focus. Wireless sensors can obtain a large amount of information by virtue of their distributed characteristics and are widely used in various fields of social production. Wireless network sensors were first used in the military field. Because of their strong concealment, they can play a role on the battlefield. They can monitor and evaluate the situation on the battlefield and can provide a scientific basis for military strategy decisions. In addition to outstanding performance in the military field, wireless sensors have also fully demonstrated their functions in the medical field. The tiny sensor is installed in the patient's body to monitor the patient's physical condition in real time. In case of emergencies, it can be discovered in time for rescue time. Environmental experts can use the data collected by sensor nodes to study the ecological environment to monitor and track the migration of migratory birds and wild animal life, to monitor the forest environment and fire warning, as well as problems that occur during the mining of mineral resources, such as gas concentration monitoring and water seepage conditions.

## 4. New Progress Experiment on Artificial Intelligence Algorithms for Big Data Processing of IOT Systems on Intelligent Production Lines

### 4.1. Experimental Hardware

This study discusses the operation of the Internet of Things system in artificial intelligence algorithms, especially when the Internet of Things technology is needed. Different specifications of the system hardware will also have different effects on the experiment.  Table 1 is the hardware parameters of this experiment.

According to the data in Table 2, the storage of the server used in this experiment is 16 TB, the RAM is 64 GB, the network card standard is 2 Gbps, the CPU is 4 cores, and the hard disk is 2 TB.

### 4.2. Detector Performance Parameters

There are many different types of artificial intelligence algorithms, in order to find the best intelligent algorithm, we need to detect and analyze each algorithm.  Table 3 lists the comparison data of specific artificial intelligence algorithms.

### 4.3. Simulation Environment Parameters

Different algorithms have different functions. In the specific experiment process, performance analysis and evaluation of different types of algorithms are required to ensure the authenticity of the data. The simulation parameters used in this article are all obtained based on the data of real nodes in the IOT experimental platform. The specific conditions are as follows:

According to the data in Table 4, the parameters of this IOT experimental platform are as follows: the transmitting and receiving energy consumption is 50 nJ/bit, the number of nodes is less than 100, the initial node energy is 0.7 J, the time slice is 32 s, the voltage is 3.6 V, the data queue length is 15 kb, the transmission distance is greater than 20 m, the data processing energy consumption is 7 nJ/bit, the node data transmission rate is 130 kb/s, and the area is 200 m ^*∗*^ 200 m.

**Figure 6:**
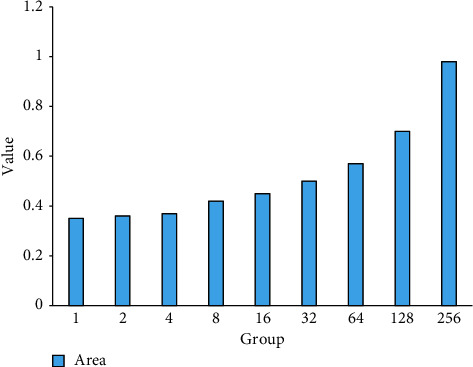
Output parallelism of computing unit.

According to the data in Figure 6, during the transition from 1 to 256 parallel output degrees, the normalized area of the computing unit is getting higher and higher. When the parallel output degree is 1, the normalized area is 0.35, and when the parallel output degree is 2, the normalized area is 0.36. When the parallel output degree is 4, the normalized area is 0.37, when the parallel output degree is 8, the normalized area is 0.42, and when the parallel output degree is 16, the normalized area is 0.45. When the parallel output degree is 32, the normalized area is 0.5, when the parallel output degree is 64, the normalized area is 0.57, and when the parallel output degree is 128, the normalized area is 0.7. When the parallel output degree is 256, the normalized area is 0.98. These data show that the shared analog-to-digital conversion circuit can indeed effectively reduce the area of the computing array.

## 5. New Developments in Artificial Intelligence Algorithms for Big Data Processing of IOT Systems on Intelligent Production Lines

### 5.1. Data Reading Performance Analysis

In the actual use process, data sources may be diverse, there are multiple copies in each metadata, and any copy can send a metadata read request in response to the request of the client.

**Figure 7:**
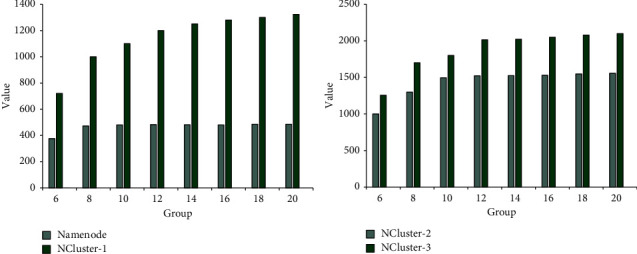
Data reading performance analysis.

According to the data in Figure 7, four different copies were used for data reading and analysis in this experiment. During the reading process, the client is in an increasing state. According to the specific data, the data processing of Namenode copy is very general. When the number of clients is 6, 376 data are processed, when the number of clients is 8, 473 data are processed 473, and when the number of clients is 10, 480 data are processed. When the number of clients is 12, 483 data are processed, when the number of clients is 14, 482 data are processed, when the number of clients is 16, 481 data are processed, when the number of clients is 18, 486 data are processed, and when the number of clients is 20, 485 data are processed. According to this data, it can be seen that the Namenode mode reads data very poorly. When the number of clients reaches 8, the data processing basically remains unchanged. When the NCluster-1 system is used for data reading, the data processing speed is improved, and as the number of clients increases, the reading speed becomes faster and faster. When the number of clients is 6, 720 data are processed. When the number is 20, 1323 data are processed, but according to specific data, when the number of clients reaches 12, the data reading speed of the NCluster-1 system will remain stable. The NCluster-2 system is used for data reading, when the number of clients is 6, 1000 data are processed, and when the number of clients is 20, 1557 data are processed. The NCluster-3 system is used for data reading, when the number of clients is 6, 1256 data are processed, when the number of clients is 20, 2100 data are processed, and the NCluster system will remain stable when the number of clients reaches 12.

**Figure 8:**
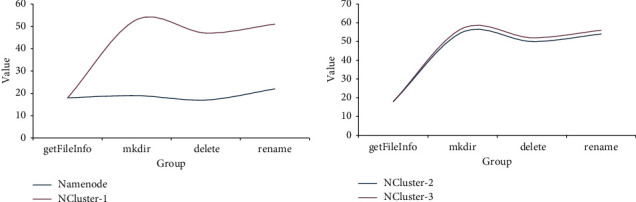
Data time-consuming comparative analysis.

According to the data in Figure 8, when the Namenode mode is used for data reading, the creation time is 18 ms, the running time is 19 ms, the deletion time is 17 ms, and the output result time is 22 ms. According to the data, the completion times of the four operations in the Namenode mode are relatively close. When using NCluster-1 mode for data reading, the creation time is 18 ms, the running time is 53 ms, the deletion time is 47 ms, and the output result time is 51 ms. When using NCluster-2 mode for data reading, the creation time is 18 ms, the running time is 55 ms, the deletion time is 50 ms, and the output result time is 54 ms. When using NCluster-3 mode for data reading, the creation time is 18 ms, the running time is 57 ms, the deletion time is 52 ms, and the output result time is 56 ms. According to the experimental data, when operating in NCluster mode, the running, deleting, and outputting times of the system have increased significantly, and there is no obvious difference between the creation time and the Namenode mode.

### 5.2. Big Data Processing Optimization Analysis

At present, big data is in a state of explosive growth, and big data has penetrated all aspects of daily life. For example, shopping websites that commonly used in daily life can recommend products to customers according to their preferences, saving time and improving user experience. In actual use, the performance of different modes is different.

**Figure 9:**
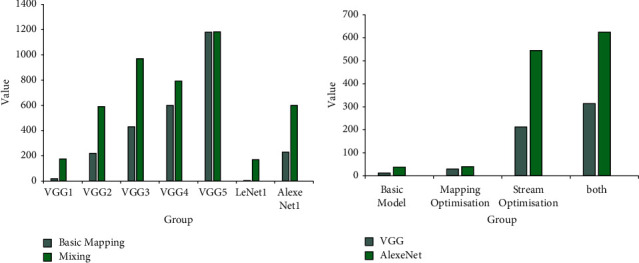
Big data processing optimization analysis.

According to the data in Figure 9, in the big data processing process, the data processing efficiency of different mapping methods is also different. The basic mapping of VGG1 is 20 Gop/s, and the hybrid mapping is 175 Gop/s. The basic mapping of VGG2 is 220 Gop/s, and the hybrid mapping is 590 Gop/s. The basic mapping of VGG3 is 430 Gop/s, and the hybrid mapping is 970 Gop/s. The basic mapping of VGG4 is 600 Gop/s, and the hybrid mapping is 793 Gop/s. The basic mapping of VGG5 is 1180 Gop/s, and the hybrid mapping is 1183 Gop/s. The basic mapping of LeNet1 is 5 Gop/s, and the hybrid mapping is 170 Gop/s. The basic mapping of AlexeNet1 is 230 Gop/s, and the hybrid mapping is 600 Gop/s. According to the data, the performance of hybrid mapping in all modes is higher than that of basic mapping. The performance of VGG in the basic mode is 13 Gop/s, the mapping performance is 30 Gop/s, and the pipeline optimization performance is 231 Gop/s. The performance of AlexeNet in the basic mode is 38 Gop/s, the mapping performance is 40 Gop/s, and the pipeline optimization performance is 545 Gop/s. According to the data, the performance of the AlexeNet mode is better than that of the VGG mode.

### 5.3. Data Access Analysis

**Figure 10:**
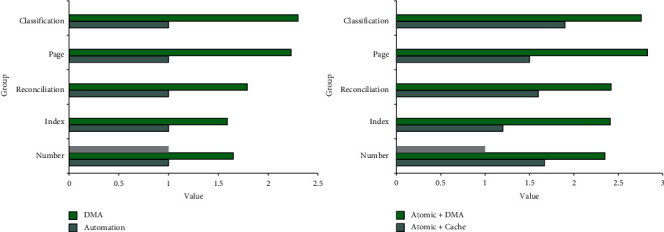
Analysis of data operation patterns.

In the process of data access, the physical address and virtual address need to be converted, and there will be a large search loss during the operation. In order to determine the specific conversion situation, this article analyzes the different access modes. The details are as follows:

According to the data in Figure 10, the performance of the system is different in different operating modes. In automation mode, the system's thesaurus performance can be improved by 1 time, the index performance of the system has been improved by 1 time, the adjustment performance of the system has been improved by 1 time, the page performance of the system has been improved by 1 time, and the classification of the system performance has been doubled. In the DMA mode, the system's thesaurus performance can be improved by 1.65 times, the index performance of the system is improved by 1.59 times, the adjustment performance of the system is improved by 1.79 times, and the page performance of the system is improved by 2.23 times, and the system classification performance has been improved by 2.3 times. In atomic + cache mode, the system's thesaurus performance can be improved by 1.67 times, the system's exponential performance by 1.2 times, the system's adjustment performance by 1.6 times, the system's page performance by 1.5 times, and the classification performance has been improved by 1.9 times. In the atomic + DMA mode, the system's thesaurus performance can be improved by 2.35 times, the system's exponential performance by 2.41 times, the system's adjustment performance by 2.42 times, the system's page performance by 2.83 times, and the classification performance has been improved by 2.76 times.

## 6. Conclusions

The rapid development of China's economy has led to the development of science and technology, and artificial intelligence technology is the focus of current research. Artificial intelligence technology can help human beings enter into unknown areas to explore, expand the scope of knowledge, improve the speed and accuracy of human work, make full use of existing data, and complete the work better. However, due to the short development time of artificial intelligence technology, there are some shortcomings in the actual application process. Therefore, this article aims to study the new development of artificial intelligence algorithms for big data processing in the Internet of Things system on intelligent production lines. It is hoped that through the development of intelligent production lines and big data processing technologies, a way to optimize artificial intelligence algorithms can be found. Although some progress has been made, there are still some shortcomings: (1) this article aims to use the Internet of Things technology to deal with big data problems, but the data security issues are not considered in the actual application process. (2) The application field needs to be further broadened. Function optimization is only one aspect of the optimization field and solving different problems requires readjusting the model and parameters of the algorithm.

## Figures and Tables

**Table 1:** System parameters.

Systems	Memory (GB)	Hard disk (GB)	Mainframe
Domain controller	3	30	2 core
Attendance system operating environment	3	30	1 core
Vcenter server	6	30	2 cores
Database server	3	30	2 cores
ESXI server	3	30	2 cores

**Table 2:** Experimental environment configuration table.

Configuration items		Parameters
Servers	64 GB	64 GB
	2 Gbps	2 Gbps
	4 cores	4 cores
	2 TB	2 TB
	64 bit	64 bit

Storage	16 TB	16 TB

**Table 3:** Comparison of detection algorithms.

Algorithms	Network	Parameters (MB)	mAP
RefineDet	VGG-16	138	82
Faster-RCNN	Resnet-50	122	77
EHN-B0	HourglassNet-B0	14	76
EHN-B3	HourglassNet-B3	36	83
StairNet	VGG-16	135	80

**Table 4:** Simulation environment parameters.

Name	Value	Name	Value
Energy consumption for transmitting and receiving	50 nJ/bit	Data queue length	15 kb
Number of nodes	Less than 100	Transmission distance	Greater than 20 m
Initial energy of nodes	0.7 J	Data processing energy consumption	7 nJ/bit
Time slice length	32 s	Node data transfer rate	130 kb/s
Voltage	3.6 V	Area	200 m ^*∗*^ 200 m

## Data Availability

Data sharing is not applicable to this article as no new data were created or analyzed in this study.
